# Neutrophil Recruitment in Pneumococcal Pneumonia

**DOI:** 10.3389/fcimb.2022.894644

**Published:** 2022-05-13

**Authors:** Catherine S. Palmer, Jacqueline M. Kimmey

**Affiliations:** Department of Microbiology and Environmental Toxicology, University of California, Santa Cruz, Santa Cruz, CA, United States

**Keywords:** neutrophil, pneumonia, *Streptococcus pneumoniae* (pneumococcus), lung, migration

## Abstract

*Streptococcus pneumoniae (Spn)* is the primary agent of community-acquired pneumonia. Neutrophils are innate immune cells that are essential for bacterial clearance during pneumococcal pneumonia but can also do harm to host tissue. Neutrophil migration in pneumococcal pneumonia is therefore a major determinant of host disease outcomes. During *Spn* infection, detection of the bacterium leads to an increase in proinflammatory signals and subsequent expression of integrins and ligands on both the neutrophil as well as endothelial and epithelial cells. These integrins and ligands mediate the tethering and migration of the neutrophil from the bloodstream to the site of infection. A gradient of host-derived and bacterial-derived chemoattractants contribute to targeted movement of neutrophils. During pneumococcal pneumonia, neutrophils are rapidly recruited to the pulmonary space, but studies show that some of the canonical neutrophil migratory machinery is dispensable. Investigation of neutrophil migration is necessary for us to understand the dynamics of pneumococcal infection. Here, we summarize what is known about the pathways that lead to migration of the neutrophil from the capillaries to the lung during pneumococcal infection.

## Introduction

*Streptococcus pneumoniae* (*Spn*) is a Gram-positive bacterium and regular member of the microbiota in the upper respiratory tract of about 0-40% of adults (Esposito et al., 2016; Smith et al., 2020) and 27-65% of children (Weiser et al., 2018). Though usually an asymptomatic colonizer in the upper respiratory tract, *Spn* can also cause clinical syndromes including pneumonia, bacteremia and meningitis. Pneumonia is the most common outcome of *Spn* infection (Brooks and Mias, 2018) and can cause disruption of lung integrity, leading to further invasion of the pathogen to the bloodstream and brain, causing bacteremia and meningitis, respectively. Although the severity of infection depends partly on the specific strain and serotype of *Spn* (Melin et al., 2010; Hyams et al., 2013), the host’s immune response also plays a critical role in pathogenesis and disease outcome. Initial detection of *Spn* in the lung is mediated by resident alveolar macrophages (Cole et al., 2014; Dockrell and Brown, 2015) and epithelial cells (Yamamoto et al., 2014), leading to significant infiltration of neutrophils into the lung. Recent work has also shown the importance of complement (Agarwal and Blom, 2015) and T cells (Ivanov et al., 2014) in modulating the innate immune response to *Spn* in the lung (Kadioglu and Andrew, 2004). Following infection, a rapid return to homeostasis and resolution of inflammation is crucial for host outcome (Kumar, 2020).

A hallmark of pneumococcal pneumonia is the rapid influx of neutrophils which play a crucial role in controlling *Spn* burden. Neutrophils can kill *Spn* intracellularly through phagocytosis but are better recognized for their diverse and highly specialized extracellular antimicrobial defenses mediated by degranulation (Amulic et al., 2012). Degranulation is a regulated process by which activated neutrophils release effectors, including cationic antimicrobial peptides, serine proteases, myeloperoxidase, and reactive oxygen species (ROS) (Effah et al., 2021). Neutrophils can also release DNA to trap bacteria in neutrophil extracellular traps (NETs) through a process known as NETosis (Effah et al., 2021). Finally, neutrophils play a critical role in regulation of inflammation through the production of cytokines which recruit additional leukocytes to the site of infection (Tecchio et al., 2014). These defenses can be very effective against *Spn* and are critical for control of symptomatic infection. Neutropenic patients are at increased risk for pneumonia (Rolston, 2001) and patients with a deficiency in degranulation experience recurrent infection and have a diminished ability to kill *Spn* (Ganz et al., 1988), a finding recapitulated in mouse models (Borsa et al., 2019).

However, like most successful pathogens, *Spn* has evolved myriad strategies to evade neutrophil-mediated immunity, which we will highlight in this review. Furthermore, because neutrophil defenses occur largely through the release of highly damaging compounds into the extracellular milieu, neutrophilic inflammation almost always causes immunopathology which can increase disease severity. Neutrophil recruitment and activity must be tightly controlled. Significant evidence exists indicating these two features can be coupled - the signals neutrophils receive during recruitment can influence effector functions at the site of infection. This review centers on the first phenomenon - mechanisms of neutrophil recruitment during pulmonary *Spn* infection.

## Neutrophil Dynamics in Murine Models

Murine models have been a powerful tool to understand the dynamics of neutrophil recruitment and will be the focus of this review, though it is important to note that all findings in murine systems must be validated to assess relevance to human disease. Pulmonary infection can be established through intranasal or intratracheal inoculation of *Spn* (Jeong et al., 2011). Pathogenesis is dependent on the bacterial strain (Mizrachi-Nebenzahl et al., 2003; Melin et al., 2010; Seyoum et al., 2011; Naucler et al., 2013; Paton and Trappetti, 2019) and is highly influenced by murine background (Gingles et al., 2001; Ripoll et al., 2010), age (Boyd et al., 2012; Williams et al., 2015; Janesch et al., 2018), and sex (Kadioglu et al., 2011a). Neutrophil infiltration into the lung occurs within the first day of infection and the infection is typically resolved (either by clearance of bacteria or death of mice) within a week. An ~100 fold increase in pulmonary neutrophils occurs following infection with *Spn* TIGR4, peaking at 18 hours post-infection (Bou Ghanem et al., 2015). The early recruitment of neutrophils is critical to bacterial clearance and host survival. Delays in recruitment lead to increased bacterial loads in the lung (Nieminen et al., 2008; Kadioglu et al., 2011b) and systemic neutrophil depletion results in higher bacterial burden and increased mortality (McNamee and Harmsen, 2006; Bou Ghanem et al., 2015). Although neutrophil influx is often correlated with neutrophil function in pneumococcal pneumonia, the link between these two phenomena requires more robust research.

Prolonged or excessive neutrophil recruitment, however, can cause damage to the pulmonary barrier and increase *Spn* invasion into the blood (Domon and Terao, 2021). In contrast to the detrimental effect of neutrophil depletion at the onset of infection, a protective effect is observed if neutrophil depletion is initiated at 18 hours post-infection, when neutrophil recruitment has already reached its peak. Depletion at this point reduces bacterial burden in the blood and increases survival (Bou Ghanem et al., 2015). Thus, damage caused by uncontrolled neutrophil inflammation can outweigh the antimicrobial benefit of the neutrophils and therefore must be tightly regulated. IL-10 is a potent anti-inflammatory signal responsible for modulation of inflammation. IL-10^-/-^ mice have increased expression of proinflammatory cytokines, exacerbated recruitment of neutrophils to the lungs, and increased susceptibility to *Spn* infection (Peñaloza et al., 2015). The regulation and dynamics of neutrophils recruitment to the pulmonary space is therefore a critical determinant of outcome following pneumococcal pneumonia.

## Recognition of *Spn*

To initiate neutrophil recruitment, tissue-resident immune cells and pulmonary epithelial cells recognize pneumococcal components by pattern recognition receptors (PRRs) such as Toll-Like Receptors (TLRs) and NOD-Like Receptors (NLRs) which results in production of cytokine and chemokines (reviewed extensively (Calbo and Garau, 2010; Hartl et al., 2018; Koppe et al., 2012; MacCain and Tuomanen, 2020; Paterson and Mitchell, 2006). A major downstream output of PRR signaling is the activation of the master transcription regulator NF-κB, which is required for optimal recruitment of neutrophils during *Spn* infection (Alcamo et al., 2001). Mice lacking TLR-adaptor MyD88 have defects in neutrophil recruitment to the lung (Albiger et al., 2005), indicating TLR signaling is important for detection of Spn. Of the 12 TLRs that exist in mice, TLR1, TLR2, TLR3, TLR4, TLR7, TLR9, and TLR13 have been shown to detect *Spn* ligands (Branger et al., 2004; Schmeck et al., 2006; Spelmink et al., 2016; Famà et al., 2020). However, not all have a significant impact on neutrophil influx (Craig et al., 2009). Knockout of TLR2 (Knapp et al., 2004; Dessing et al., 2008a) leads to defects in neutrophil recruitment, while knockout of TLR9 (Albiger et al., 2007) or TLR4 does not (Branger et al., 2004; Srivastava et al., 2005; Dessing et al., 2008b). Triple knockout mice lacking TLR7/9/13 have defects in generation of neutrophil-attracting chemokines and increased susceptibility almost as severe as MyD88^-/-^ mice. However, single deletion is not sufficient to cause susceptibility demonstrating these sensors show functional compensation *in vivo* (Famà et al., 2020). Another important family of PRR activated by *Spn* is cytosolic NOD1 and NOD2 that detect peptidoglycan (Zheng et al., 2018). Deletion of either does not affect neutrophil influx but does lead to bacterial burden increase in some tissues (Lysenko et al., 2007; Davis et al., 2011).

In addition to activation of PRRs, bacterial ligands can also serve as direct chemoattractants for neutrophils (Bloes et al., 2015). Neutrophils can sense N-formyl peptides (fMLP) produced by *Spn via* fMLP receptor. Administration of fMLP receptor antagonist prior to *Spn* infection decreased neutrophils in bronchoalveolar lavage (BALF) (Fillion et al., 2001). Pneumolysin, a major virulence factor produced by *Spn*, also has myriad effects on neutrophil migration. Recombinant pneumolysin is sufficient to induce neutrophil migration in transwell assays, demonstrating it can serve as a chemoattractant (Moreland and Bailey, 2006). Recent work has demonstrated that pneumolysin-induced neutrophil migration is mediated through the formation of pores by pneumolysin and subsequent generation of inflammatory lipids which act as chemoattractants (Adams et al., 2020). Additionally, pneumolysin is a pore-forming toxin and causes damage-induced inflammation *in vivo* (Rubins et al., 1992; Rubins et al., 1993; Rayner et al., 1995; Witzenrath et al., 2006; García-Suárez et al., 2007). Pulmonary inoculation of recombinant pneumolysin is sufficient to cause neutrophil influx *in vivo* and *Spn* lacking pneumolysin results in decreased neutrophil infiltration. However, as pneumolysin is a crucial virulence factor, this attenuated strain is rapidly cleared in many animal models which likely contributes to decreased inflammation (Rubins et al., 1995; Kadioglu et al., 2000).

## Migration of Neutrophils to the Lung

**Figure 1:**
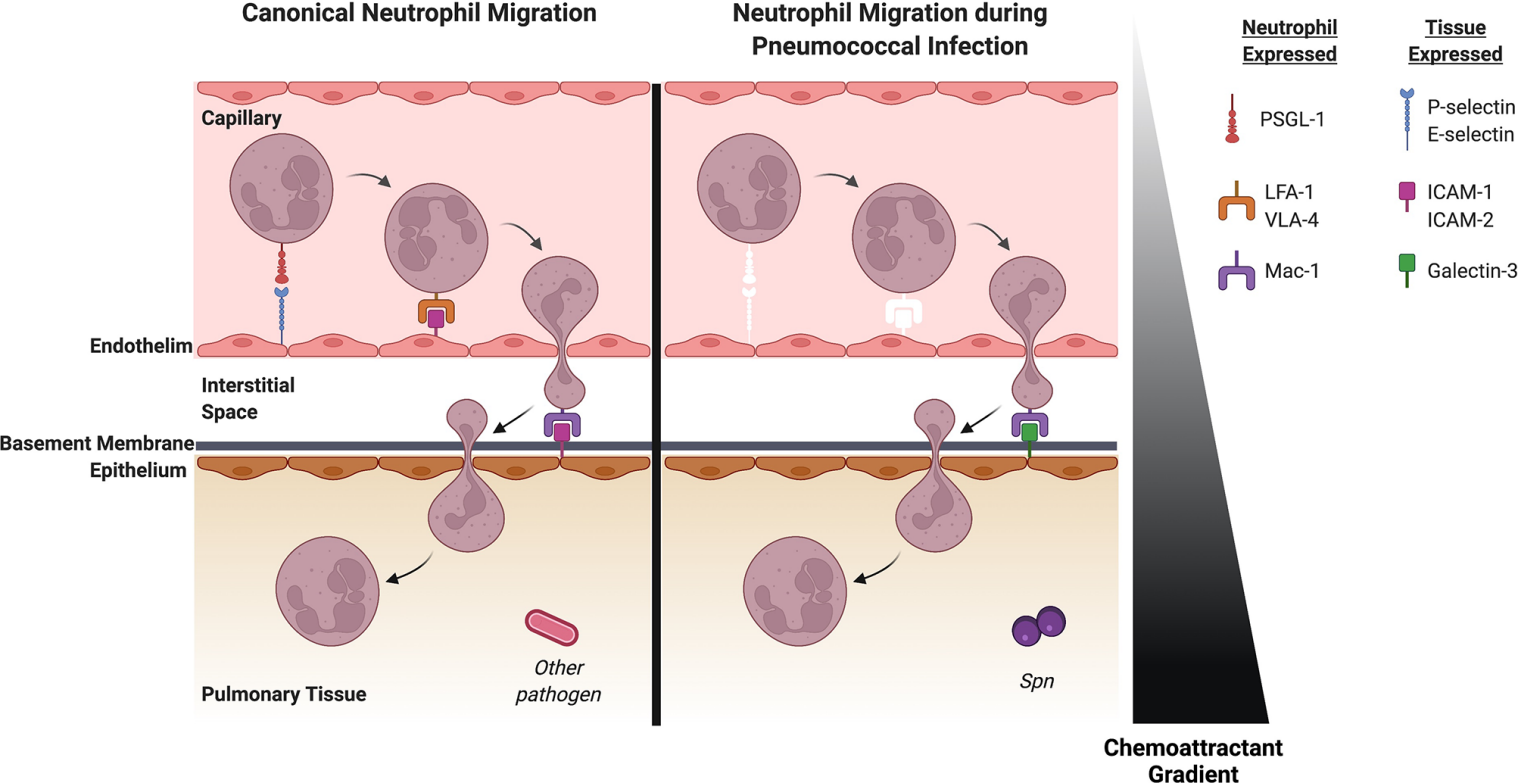
Neutrophil recruitment in the lung during pneumococcal pneumonia differs from canonical neutrophil recruitment to the lung. Left: Canonical neutrophil recruitment into pulmonary tissue. Selectins impart weak interactions between the endothelium and neutrophil which allows tethering by the integrin PSGL-1. LFA-1 and VLA-4 on the neutrophil then interact with ICAM-1 and ICAM-2 on the endothelial surface allowing the neutrophil to cross the endothelium. ICAM-1 and ICAM-2 also allows the neutrophil to bind to the epithelium *via* the integrin Mac-1 and subsequently migrate into the pulmonary space. Right: Neutrophil recruitment to the lung during Spn infection. White molecules represent factors that are important for canonical neutrophil recruitment but do not play a role in pneumococcal pneumonia. Selectins are dispensable for neutrophil recruitment for pneumococcal pneumonia. Mechanical entrapment in the thinner vessels likely plays a role in initial attraction of the neutrophil to the endothelium. The canonical integrin-ligand pairs that allow the neutrophil to bind to the endothelium seem to be non-essential in pneumococcal pneumonia, so unknown ligands and integrins on the endothelium and the neutrophil may play a role here. Once the neutrophil is in the interstitial space, ligands on the epithelium capture the neutrophil. Although canonical ligands like ICAM-1 and ICAM-2 have been shown to be indispensable here, Galectin-3 has a significant role in subsequent movement of the neutrophil into the pulmonary space. *Spn, Streptococcus pneumoniae.* Created with BioRender.com.

Neutrophils are bone marrow-derived cells that circulate in the bloodstream until recruited into tissue. Inflammation induces vasodilation to slow blood flow, allowing neutrophils to sequentially migrate through the endothelium, interstitium, basement membrane and the epithelium (**Figure 1**). Each of these steps is orchestrated through interactions between the neutrophil and membrane-bound ligands or soluble factors (Adams et al., 2021). Some chemoattractants are known to play a role in specific movements across the endothelium, epithelium or through a specific space. For example, CD73, which plays a role in adenosine production, contributes to transendothelial but not transepithelial migration. We will highlight these nuances during our discussion of specific factors.

Transendothelial migration from the blood begins when inflammatory cytokines upregulate adhesion molecules on the endothelial surface. In most tissues, neutrophil migration occurs in post-capillary venules. Migration into the alveolar space, however, occurs in the capillaries which have a smaller diameter and it is thought that mechanical entrapment may be important for neutrophil migration (Doyle et al., 1997). Capture of neutrophils is typically mediated by E-selectin (CD62E) and P-selectin (CD62P). However, pulmonary neutrophil migration during *Spn* uniquely happens independently of these interactions (Mizgerd et al., 1996). Consistent with this, mice lacking neutrophilic P-selectin glycoprotein ligand-1 (PSGL-1), which interacts with endothelial E- and P-selectin, show no defect in pulmonary migration during *Spn* (Ramos-Sevillano et al., 2016). However, PGSL-1 knockout mice show increased bacterial burden in the lung and blood indicating PGSL-1 has other roles in the prevention of invasive disease. Bacteria lacking a protease that can degrade PGSL-1 (*Spn* Δ*zmpC)* stimulate increased neutrophil inflammation (Surewaard et al., 2013), pointing towards a potential role for PGSL-1 or similar integrins during infection. Surface expression of other adhesion molecules involved in neutrophil recruitment including CD54 (ICAM-1), CD102 (ICAM-2), CD106 (VCAM-1), and E-selectin is not upregulated on pulmonary endothelium following exposure to two different serotypes of *Spn* (Bullard et al., 1995; Moreland et al., 2004), suggesting these are not major drivers regulator of neutrophil influx during *Spn* infection.

Integrins are heterodimers consisting of an α and β subunit and are upregulated during inflammation to promote neutrophil attachment to the endothelial surface. CD18 is a β subunit that can dimerize with four different α subunits (CD11a, CD11b, CD11c, and CD11d). CD11a/CD18 (LFA-1) is canonically an important integrin for neutrophil arrest and has a known role in several bacterial pneumonias (Maas et al., 2018). However, inhibition or deletion of CD11a or CD18 does not affect neutrophil recruitment during *Spn* infection (Mizgerd et al., 1997; Mizgerd et al., 1999; Maas et al., 2018). Similarly, ICAM-1 (CD54), the canonical binding partner of CD11a/CD18, does not contribute to pulmonary recruitment during infection. ICAM-1 expression is not upregulated on pulmonary epithelium by *Spn* (Burns et al., 1994) and mutation of ICAM-1 lead to defects in peritoneal, but not pulmonary recruitment during *Spn* infection (Bullard et al., 1995). Therefore, ICAM-1 plays a tissue-specific role in neutrophil recruitment. Another β integrin, very-late antigen 4 (VLA-4), is increased on neutrophils during *in vitro Spn* infection (Kubes et al., 1995). Studies in mice, however, did not observe this increase and antibody blockade of VLA-4 has no effect during *Spn* infection (Tasaka et al., 2002). Redundancy in adhesion molecules likely contributes, at least in part, to modest phenotypes in recruitment defects. However, a double-mutant lacking both P-selectin and ICAM-1 still shows no defect in *Spn*-mediated neutrophil recruitment (Bullard et al., 1995) indicating mechanisms of neutrophil recruitment are unique to *Spn* compared to other bacterial pneumonias.

Following sequestration on the endothelial surface, neutrophil movement is canonically mediated by the neutrophilic integrin L-selectin (CD62L) and gap junction protein connexin 43. Knockout of L-selectin does not affect neutrophil recruitment in response to *Spn* in mice (Doyle et al., 1997) and work in the rabbit lung associated *Spn* infection with downregulation of L-selectin on neutrophils (Burns and Doerschuk, 1994). While connexin 43 plays an important role in pneumococcal meningitis (Bello et al., 2020), it has not been well studied in pneumonia. Once the neutrophil migrates through the endothelium, it must travel through the interstitium and cross the basement membrane. Overall, these processes are not well-characterized, but attention has been brought to glycosaminoglycans as a key component of the extracellular space and as modulators of the inflammatory response (Souza-Fernandes et al., 2006). Studies in nasopharyngeal tissue implicate glycosaminoglycans in *Spn* attachment to epithelial cells (Tonnaer et al., 2006). Expression of certain glycosaminoglycans may therefore benefit the bacterium more than mediate neutrophil recruitment; however these processes are not well studied in the lung and therefore more research is needed to understand how neutrophils cross the endothelium, interstitium and basement membrane.

After crossing the basement membrane, neutrophils undergo transepithelial migration which consists of basolateral adhesion, paracellular transit and alveolar entry. Like transendothelial migration, migration of neutrophils across the epithelium is orchestrated by ligand-integrin binding. The best-characterized receptor for basolateral adhesion of neutrophils is CD11b/CD18 (Mac-1). During *Spn* infection, blockade or knockout of CD11b decreased neutrophil influx and increased bacterial lung burden (Kadioglu et al., 2011b). ICAM-1 is a ligand for CD11b/CD18 (as well as the aforementioned CD11a/CD18 endothelial transmigration integrin), but knockout or antibody blockade of ICAM-1 did not affect neutrophil infiltration (Moreland et al., 2004; Kadioglu et al., 2011b). The role of ICAM-1 in canonical transepithelial and transendothelial migration has been reported, but this ligand does not seem to be necessary for pneumococcal pneumonia. Instead, galectin-3 is a ligand for CD11b/CD18 that is upregulated in lungs infected with *Spn* but not other bacterial species (Nieminen et al., 2008). Galectin-3 deficient mice showed decreased neutrophil influx and increased bacterial load in response to pneumococcal pneumonia, indicating galectin-3 is a *Spn*-specific integrin that aids in migration of neutrophils.

To cross the epithelial barrier during paracellular transit, neutrophils release serine proteases and matrix metalloproteases that degrade intercellular junctions. Neutrophil elastase, cathepsin G and proteinase 3 can all degrade junction proteins such as E-cadherin. Recent work in human lung tissue showed that pneumococcal infection reduced the junction components occludin, ZO-1, claudin-5 and VE-cadherin but did not change the presence of other claudins (Peter et al., 2017). Pneumolysin also increases the permeability of the pulmonary lining through disruption of intercellular junctions (Rubins et al., 1993; Rayner et al., 1995; Knippenberg et al., 2015) which may allow neutrophils to cross the epithelium more easily. Once neutrophils pass the intercellular junctions of the epithelium, they interact with the apical side of the epithelium and enter the pulmonary space.

## Chemokines and Cytokines

Neutrophil migration is ultimately accomplished through the cooperation of integrin-ligand interactions and directed migration down a gradient of intermediate-target and end-target attractants. The gradient of chemokines, inflammatory lipids, serum proteins and bacterial components induce migration through activation of G-protein coupled receptors (GPCRs) on neutrophils. Intermediate-target attractants control directed neutrophil migration *en route* to the site of inflammation, whereas end-target attractants are preferred by neutrophils and determine local neutrophil activity once in the lung.

Chemokines are important intermediate-target chemoattractants that interact with the neutrophil *en route* to the lung. In mice, the chemokines CXCL1 (KC), CXCL2 (MIP-2), and CXCL5 (LIX) mediate the neutrophils’ basolateral adhesion to the lung epithelium (Adams et al., 2021). CXCL1 and CXCL2 are produced primarily by myeloid cells (Fillion et al., 2001; Craig et al., 2009), whereas epithelial cells are the predominant sources of CXCL5 (Yamamoto et al., 2014).

CXCL1 is produced in response to TLR-mediated NF-κB activation (Paudel et al., 2019) and plays a crucial role in the recruitment of neutrophils (McColl and Clark-Lewis, 1999) after *Spn.* Mice lacking CXCL1 showed decreased neutrophil abundance in the BALF as well as increased bacterial burden in the lung, blood and BALF. CXCL1 is also required for emergency granulopoiesis during *Spn* infection, which also contributes to decreased neutrophil numbers recruited to the lungs (Paudel et al., 2019). Once neutrophils reach the pulmonary space, they make CXCL2 upon stimulation by local cues produced by myeloid and epithelial cells (Kamata et al., 2016). CXCL2 is canonically a potent chemoattractant in bacterial pneumonia (Adams et al., 2021), but its role has not been well-studied with *Spn*. One study showed that the administration of the proinflammatory cytokine IL-12 could improve innate defense in the lung against *Spn* by inducing IFN-γ production, enhancing CXCL2 expression, and thereby increasing neutrophil recruitment to the lung after infection (Sun et al., 2007). This suggests a direct role for CXCL2 in neutrophil migration during pneumococcal pneumonia.

In addition to the myeloid-derived CXCL1 and CXCL2, epithelial-derived CXCL5 enhances neutrophil recruitment (Gibbs et al., 2014; Yamamoto et al., 2014). Mice with previous *Spn* infection had faster bacterial clearance upon secondary infection in part to prolonged stability of the chemokine ligand CXCL5 transcripts by IL-17a (Shenoy et al., 2020) resulting in more rapid neutrophil recruitment to the lung. Mice with heightened levels of CXCL5 had increased neutrophil influx, better control of pulmonary burden and increased survival upon *Spn* infection (Mancuso et al., 2018).

CXCL1, CXCL2 and CXCL5 all bind to CXCR1 and CXCR2 receptors on neutrophils and therefore these chemokines and receptors are emerging as exciting therapeutic targets to control neutrophil influx (Planagumà et al., 2015; Sundaresh et al., 2021). The role of CXCR1 has not been explored in pneumococcal pneumonia, but CXCR2 is important for optimal bacterial clearance. *Spn* infection of CXCR2 knockout mice or mice given a CXCR2 antagonist resulted in a defect in neutrophil influx and an associated increase in bacterial counts in BALF and lungs (Eash et al., 2010; Herbold et al., 2010).

Production of proinflammatory cytokines such as TNF-α, IL-1, and IL-17 are also important for neutrophil regulation (Bergeron et al., 1998). Antibody blockade (Takashima et al., 1997) or knockout (Jeong et al., 2015) of TNF-α lead to increased bacterial burden and mortality, though it did not directly affect neutrophil recruitment. Mice with defects in IL-1β but not IL-1α showed significantly worse immunopathology, bacterial burden and mortality following pulmonary infection with *Spn* (Kafka et al., 2008). A triple mouse mutant deficient in TNF-α receptors (TNFR1, TNFR2) and IL-1 receptor (IL-1RI) also had reduced neutrophil influx to the lung (Jones et al., 2005). The cytokine IL-17, typically associated with neutrophilic responses, acts in concert with TNF-α and IL-1 to promote inflammation. IL-17A can be either beneficial or detrimental depending on the strain of *Spn*, a process attributed to the abundance and function of recruited neutrophils (Ritchie et al., 2018). For example, mice lacking IL-17a had decreased neutrophil influx following infection with several strains of *Spn*, but had strain-dependent correlation with mouse survival. Together proinflammatory cytokines influence neutrophil migration at multiple levels including activation of myeloid cell effector function, upregulation of epithelial, endothelial, and neutrophil adhesion molecules, and activation of neutrophil extravasation. As such, the contribution of each of these factors may not directly reflect on neutrophil numbers, but interruption of their signaling may alter neutrophil migration and function.

## Non-Chemokine Chemoattractants

Cleavage or processing of host molecules like extracellular matrix, complement proteins and phospholipids also produces a variety of chemoattractants. Matrikines are degraded components of the extracellular matrix that can act as intermediate chemoattractants for neutrophils during pulmonary inflammation (Adams et al., 2021). These components have not been well-studied in pneumococcal infection, but producers of the canonical matrikine proline-glycine-proline (PGP) are upregulated in response to *Spn* (Akthar et al., 2015). Activation of the host complement system generates classical neutrophil chemoattractants including the end-target attractant C3a and C5a, which increase in lungs during *Spn* infection (Angel et al., 1994; Paterson and Mitchell, 2006). The role of C3a has not been directly tested during *Spn*, but C5a neutralization did not affect neutrophil numbers in BALF (Müller-Redetzky et al., 2020) suggesting complement is not a main driver of neutrophil influx.

Inflammatory lipids are another important mediator which are produced upon phospholipid cleavage by phospholipase A_2_ to generate arachidonic acid (Rubins et al., 1994). Arachidonic acid modification by lipoxygenases produces chemoattractants leukotriene B4 (LTB_4_) and hepoxilin A3 (HXA_3_) (Dobrian et al., 2011). LTB_4_ is an intermediate stage chemoattractant produced by the lipoxygenase 5-LOX and is canonically important for transepithelial migration (Palmblad et al., 1981). Mice lacking 5-LOX exhibited increased susceptibility to pneumococcal pneumonia which could be reversed by exogenous administration of LTB_4_ (Mancuso et al., 2010). Interestingly, the pneumococcal virulence factor pneumolysin increases activity of phospholipase A_2_ and production of LTB_4_ in human neutrophils (Cockeran et al., 2001a; Cockeran et al., 2001b; Bhowmick et al., 2012; Bhowmick et al., 2017). HXA_3_ is another phospholipase A_2_-derived end-target chemoattractant that acts mostly on the apical side of the lung epithelium. HXA_3_ robustly attracts neutrophils across the lung epithelium during *Spn* infection (Bhowmick et al., 2013; Adams et al., 2020) and induced pulmonary inflammation (Bhowmick et al., 2017).

Another important host-derived modulator of neutrophil function is purinergic signaling. Purine nucleotides such as ATP, ADP, and adenosine differentially activate purinergic receptors including P2X (activated by ATP), P2Y (activated by ATP and ADP) and P1 receptors (activated by adenosine) (Huang et al., 2021). In homeostatic conditions, extracellular ATP is minimal due to rapid hydrolysis by surface associated nucleases including CD39 (converts ATP to AMP) and CD73 (converts AMP to adenosine), and extracellular nucleosides (degrade adenosine) (Baron et al., 2015). During inflammation, extracellular ATP can be acutely increased by secretion *via* connexins or pannexins (Muñoz et al., 2021) or upon loss of membrane integrity due to cell death. In the context of *Spn*, the virulence factor pneumolysin colocalizes with and transcriptionally upregulates P2X_7_R on neutrophils (Domon et al., 2016). A separate study (Cuypers et al., 2020) showed that experimental augmentation of extracellular ATP can protect from pneumolysin-induced neutrophil degranulation, though the effect was not dependent on P2X_7_R, suggesting a role for other P2 receptors or downstream signaling. For example, extracellular adenosine contributes to bacterial clearance and survival of mice following *Spn* infection. This effect is mediated in part by CD73-dependent generation of adenosine from AMP and can be pharmacologically augmented by preventing adenosine degradation (Bou Ghanem et al., 2015). Additionally, the role of CD73-dependent neutrophil attraction is specific to transendothelial migration and does not have a role in transepithelial migration. The mechanism of adenosine-based protection is not well-defined in *Spn*, but several P1 adenosine receptors (A1, A2B, A3) have immunomodulatory roles in endotoxin-induced pulmonary inflammation, and thus remains an open area for exploration during Spn infection (Ngamsri et al., 2010; Schingnitz et al., 2010; Wagner et al., 2010).

## Discussion

Neutrophils are critical mediators of pneumococcal pneumonia and their rapid influx from the bloodstream into the pulmonary space is required for clearance of *Spn*. Once recruited to the lungs, neutrophils produce proinflammatory cytokines, perform phagocytosis, NETosis or degranulation and can cause considerable damage to host tissue if infection is not resolved in a reasonable time. Therefore, neutrophil influx to the lung is a critical factor in pneumococcal pneumonia pathogenesis.

The process of directed neutrophil migration from the blood across the endothelial and epithelial barriers depends on integrin-ligand interactions between the neutrophil and barrier cells as well as GPCR-mediated sensing of a gradient of chemoattractants. After detection of *Spn* through PRRs, resident myeloid cells and epithelial cells generate cytokines that signal to endothelial and epithelial cells to upregulate ligands with which to attract and tether neutrophils. The canonical neutrophil chemokines in mice, CXCL1, CXCL2, and CXCL5 as well as other chemoattracts created by the host (C5a, C3a, LTB_4_, HXA_3_) and *Spn* (fMLP, pneumolysin), guide the neutrophil to the lung through GPCR recognition by the neutrophil and subsequent movement. Neutrophils receive cytokine signals to express integrins and produce granules that aid in migration. Some of the canonical integrins that mediate neutrophil migration are dispensable in *Spn* infection and more research is needed to characterize mediators that dictate neutrophil movement from the bloodstream (**Table 1**). In particular, the necessary ligand-integrin pairs for transendothelial migration are not known for pneumococcal pneumonia and further studies in this particular process are needed.

**Table 1:** Effect of *in vivo* depletion or knockout of key neutrophil migration mediators on neutrophil influx, survival and bacterial burden in pneumococcal pneumonia.

		*Influx*	*Host Survival*	*Burden*	
***Detection*** *TLRs*	**TLR2**	↓	NS	NS	(Branger et al., 2004; Dessing et al., 2008a).
	**TLR4**	NS	NS	↑	(Dessing et al., 2008b; Knapp et al., 2004)
	**TLR9**	NS	↓	↑	(Albiger et al., 2007)
*TLR adaptor*	**MyD88**	↓	↓	↑	(Albiger et al., 2005)
*NODs*	**NOD1**	NS	NS	NS	(Lysenko et al., 2007)
	**NOD2**	NS	–	–	(Davis et al., 2011)
	**NF-κB**	↓	–	–	(Alcamo et al., 2001)
	**TLR2 & NOD2**	–	–	↑	(Davis et al., 2011)
***Migration*** *Integrins*	**CD18/CD11**	NS	–	–	(Mizgerd et al., 1997; Mizgerd et al., 1999)
	**LFA-1**	NS	–	NS	(Kadiolglu et al., 2011b)
	**VLA-4**	NS	–	–	(Kubes et al., 1995)
	**Mac-1**	↓	↓	↑	(Ren et al., 2004; Kadioglu et al., 2011b)
*Ligands*	**E-selectin**	NS	–	–	(Mizgerd et al., 1996)
	**P-selectin**	NS	–	–	(Mizgerd et al., 1996)
	**PSGL-1**	NS	–	↑	(Ramos-Sevillano et al., 2016)
	**ICAM-1**	NS	–	↑	(Bullard et al., 1995; Kadioglu et al., 2011b)
	**VCAM-1**	NS	–	–	(Moreland et al., 2004)
	**L-selectin**	NS	–	–	(Doyle et al., 1997)
	**Galectin-3**	↓	–	↑	(Nieminen et al., 2008)
	**P-selectin & ICAM-1**	NS	–	–	(Mizgerd et al., 1996)
***Chemoattractants***	**CXCL1**	↓	↓	↑	(Paudel et al., 2019)
*Chemokines*	**CXCR2**	↓	↓	↑	(Herbold et al., 2010)
*Lipids*	**HXA_3_**	↓	↑	↓	(Bhowmick et al., 2017; Adams et al., 2020)
	**LTB_4_**	↓	–	↑	(Mancuso et al., 2010)
***Signaling*** *Cytokines*	**IL-17a**	↓	↓	↑	(Ritchie et al., 2018)
	**TNF-α**	NS	↓	↓	(Takashima et al., 1997; Jeong et al., 2015)
	**IL-10**	↑	↓	↓	(Peñaloza et al., 2015)

↓ decreased; ↑ increased; - not determined; NS, no significant difference.

Improved models of neutrophil recruitment throughout infection may help elucidate our understanding of the contribution of these immune effectors to the pathophysiology of *Spn* infection. Although murine models have allowed us to gain a better understanding of neutrophil recruitment, the application of other physiologically relevant models such as human organoids (Gkatzis et al., 2018), intravital microscopy (Alizadeh-Tabrizi et al., 2021), and humanized mice (Zheng et al., 2022) to the study of pneumococcal pneumonia will be indispensable in developing our understanding of human infection.

Furthermore, host changes such as age (Tseng and Liu, 2014), neutrophil age (Simmons et al., 2021), time of day (Scheiermann et al., 2018), and co-infection (Jochems et al., 2018) modulate neutrophil recruitment and but their contribution to the pathophysiology of *Spn* infection of the lung is not well-studied and requires more attention. For example, neutrophils in aged hosts show aberrant chemotaxis and reduced NETosis. Pneumococcal pneumonia is particularly prevalent in older patients, but the precise interactions that lead to this dysfunction require more research (Grudzinska et al., 2020). The respiratory microbiome is also an increasingly compelling contributor to pulmonary disease which may be necessary for understanding neutrophil recruitment in pneumococcal pneumonia (Li et al., 2019). Additionally, it has been increasingly recognized in the field of neutrophil biology that not all neutrophils are alike but rather that there are subpopulations of neutrophils. Definitions of N1 and N2 neutrophil subsets as pro-inflammatory and anti-inflammatory respectively have emerged out of cancer biology (Ohms et al., 2020) and these classifications have not been explored with regard to pneumococcal pneumonia but may provide insight to the complex role of neutrophils in *Spn* infection.

The precise inflammatory landscape caused by excessive recruitment of neutrophils is of interest for therapeutic reasons (Németh et al., 2020). For example, dampening neutrophil recruitment with a CXCR2 antagonist has been shown to alleviate respiratory inflammation in patients with COPD (Rennard et al., 2015). Therefore, further studies defining mediators of neutrophil recruitment that are active in pneumococcal pneumonia may allow for the development of clinical therapeutics.

## Author Contributions

CP: conceptualization and writing for original draft preparation. CP and JK: writing, review, editing, and visualization. All authors contributed to the article and approved the submitted version.

## Funding

JK received funding from UC Santa Cruz which was used to support authors’ effort in writing this manuscript as well as publication fees.

## Conflict of Interest

The authors declare that the research was conducted in the absence of any commercial or financial relationships that could be construed as a potential conflict of interest.

## Publisher’s Note

All claims expressed in this article are solely those of the authors and do not necessarily represent those of their affiliated organizations, or those of the publisher, the editors and the reviewers. Any product that may be evaluated in this article, or claim that may be made by its manufacturer, is not guaranteed or endorsed by the publisher.
